# Chromatographic and Molecular Insights into Fatty Acid Profiles of Thermophilic *Lactobacillus* Strains: Influence of Tween 80^TM^ Supplementation

**DOI:** 10.3390/molecules31010014

**Published:** 2025-12-19

**Authors:** Dorota Zaręba, Małgorzata Ziarno

**Affiliations:** 1 Professor E. Pijanowski Catering School Complex in Warsaw, 04-110 Warsaw, Poland; dorotazareba@gmail.com; 2 Department of Food Technology and Assessment, Institute of Food Science, Warsaw University of Life Sciences—SGGW (WULS—SGGW), Nowoursynowska 159c St., 02-776 Warsaw, Poland

**Keywords:** lactic acid bacteria, *Lactobacillus*, fatty acids, fatty acid profile, Tween 80^TM^

## Abstract

The molecular fatty acid (FA) profiles of seven certified thermophilic *Lactobacillus* strains, including the influence of an extracellular source of oleic acid (as Tween 80^TM^), were characterised using advanced chromatographic and spectrometric methods. Cyclic and conjugated fatty acids were identified by GC-MS using co-injections with authentic standards, ECL, and diagnostic EI ions, with a secondary confirmation using literature data. Additionally, the molecular-level characterisation of fatty acid profiles of bacterial cells was summarised using the latest analytical approaches, highlighting inconsistencies and differences reported in previous studies. Six saturated fatty acids, two single-branched fatty acids with iso and anteiso structures, one hydroxy fatty acid, nine unsaturated fatty acids with one double bond, two fatty acids with unsaturated double bonds, six fatty acids with conjugated bonds, and three fatty acids with a cyclic part in the carbon chain were identified. Within these fatty acids, we also demonstrated the limitations of molecular chromatographic resolution and structural discrimination, which impacted the effective identification of fatty acids in our research. We confirmed the significant differences in terms of the identification of C18:1,*cis-*9 and C18:1,*cis-*11 acids, as well as cycC19:0,*cis-*10,11, and cycC19:0,*cis-*9,10 acids. The observations at the molecular–physiological interface related to the lack of growth of *L. acidophilus* strains and the visibly reduced growth of *L. delbrueckii* subsp. *lactis* ATCC 4797 in the MRS without the addition of Tween 80^TM^ allowed us to confirm that the exclusion of this medium is useful in differentiating the lactobacilli.

## 1. Introduction

The fatty acid (FA) composition of lactic acid bacteria (LAB) membranes plays a fundamental role in maintaining membrane fluidity, structural integrity, and adaptability to environmental stressors. This composition is modulated by a variety of molecular and physicochemical factors, including the temperature; pH; oxygen availability; growth phase; salt concentration; and, importantly, the composition of the culture medium [1,2,3]. A common additive in culture media for LAB is Tween 80^TM^, a non-ionic surfactant composed of polyoxyethylene(20)sorbitan monooleate. As a water-soluble derivative of oleic acid (C18:1,*cis-*9), it serves both as a lipid precursor and a molecular surfactant that enhances bacterial growth, particularly among *Lactobacillus* species [1,2,3,4]. Exogenous C18:1,*cis-*9 acid, delivered through Tween 80^TM^, has been shown to significantly influence the fatty acid composition of LAB membranes. Beal et al. [5] reported that supplementation with this monounsaturated acid leads to increased levels of cyclic C19:0 fatty acids and a higher unsaturated/saturated FA ratio. This shift results from elevated concentrations of C18:1(9c) and C20:1, accompanied by a reduction in saturated FA such as C16:0 and C18:0, while levels of C18:1(11c) and cycC19:0 remained unchanged. Similarly, Partenen et al. [2] demonstrated that Tween 80^TM^ supplementation altered the membrane lipid profiles in six *Lactobacillus delbrueckii* strains, enabling their classification into three chemotaxonomic groups based on the cyclic C19:0 content. Notably, they observed the conversion of exogenous C18:1,*cis-*9 and C18:1(11c) to cycC19:0,*cis-*9,10 and cycC19:0,*cis-*10,11, respectively. In the absence of Tween 80^TM^, these cyclic derivatives were undetectable, suggesting that many LAB strains lack the intrinsic biosynthetic capacity for their synthesis [4,6,7]. Fatty acids with diverse structural features—including saturated, monounsaturated, polyunsaturated, branched, hydroxy, conjugated, and cyclic forms—collectively determine the physicochemical properties of bacterial membranes. This is particularly relevant in thermophilic *Lactobacillus* strains, where enhanced membrane plasticity is critical for survival under elevated temperatures [8]. Several studies have highlighted the association between the membrane lipid composition, including the presence of cardiolipin, glycolipids, and aminophospholipids, and cellular cryotolerance or stress resistance [8,9]. Although primarily an oleic acid donor, Tween 80™ is not chemically pure and may contain traces of other long-chain fatty acids, such as palmitic and stearic acids. These additional compounds may interfere with interpretations of membrane remodelling responses [3,9]. Therefore, understanding the nuanced biochemical impact of Tween 80™ supplementation is crucial for an accurate assessment of bacterial lipid metabolism.

The Sherlock Microbial Identification System (MIDI Inc.) remains the most frequently employed approach for identifying methylated fatty acids in Firmicutes, including *Lactobacillus* spp. [3]. However, this method offers limited resolution for structurally similar or functionally relevant fatty acids. Alternative techniques, such as the advanced chromatographic and spectrometric methods employed in this study, offer enhanced molecular resolution, enabling better differentiation of positional and geometric isomers. This capability is essential for resolving analytical challenges, such as distinguishing C18:1,*cis-*9 from C18:1,*cis-*11 or separating structurally similar cyclic fatty acids like cycC19:0,*cis-*9,10 and cycC19:0,*cis-*10,11 [3].

Accordingly, the present study aims to characterise the molecular fatty acid profiles of selected thermophilic *Lactobacillus* strains, with particular attention to the impact of C18:1,*cis-*9 supplementation via Tween 80^TM^. Moreover, this work seeks to resolve ambiguities in fatty acid identification present in the previous literature by employing high-resolution chromatographic techniques. Through this approach, we also explore the chemotaxonomic and physiological relevance of FA profile variations in LAB, aiming to expand our current understanding of bacterial lipid metabolism and adaptation mechanisms.

## 2. Results

The MRS medium is recommended in culture collection specifications and is widely used to culture LAB of the *Lactobacillus* genus. No growth of *L. acidophilus* strains was observed when the culture was incubated at 37 °C for 24 h under anaerobic conditions. The growth on the medium with Tween 80^TM^ allowed us to determine the biomass of four *L. acidophilus* strains. The remaining *Lactobacillus* strains grew in the MRS medium without Tween 80^TM^.

It must be emphasised that the LAB fatty acid profile included 29 fatty acids (Table 1 and Table 2), including Six saturated fatty acids, two single-branched acids fatty acids with iso and anteiso structures, one hydroxy fatty acid, nine unsaturated fatty acids with one double bond (monounsaturated fatty acids), two fatty acids with unsaturated double bonds (polyunsaturated fatty acids), six fatty acids with conjugated bonds, and three fatty acids with a cyclic part in the carbon chain. C14:0, C16:0, C16:1,*cis-*9, C18:0, C18:1,*cis-*9, C18:1,*cis-*11, cycC19:0,*cis-*9,10, and cycC19:0,*cis-*10,11 acids were predominant in most bacterial biomasses. According to a one-way ANOVA followed by Tukey’s post hoc test (*p* < 0.05), the most significant inter-strain differences were observed for C18:1,*cis-*9, C18:1,*cis-*11, cycC19:0,*cis-*9,10, and cycC19:0,*cis-*10,11, confirming their strong discriminative role within the tested thermophilic *Lactobacillus* strains. These acids correspond to the main differentiating factors also visible in the ratios of C18:1,*cis-*9/C18:1,*cis-*11 and cycC19:0,*cis-*9,10/cycC19:0,*cis-*10,11 (Table 1 and Table 2).

These acids are the most characteristic and stimulate LAB growth. The separation of C18:1,*cis-*9 acid from C18:1,*cis-*11 acid and cycC19:0,*cis-*9,10 acid from cycC19:0,*cis-*10,11 acid and their identification are the most common problems reported in the literature. This often leads to incorrect identifications and many generalisations regarding the content of these acids.

**Figure 1:**
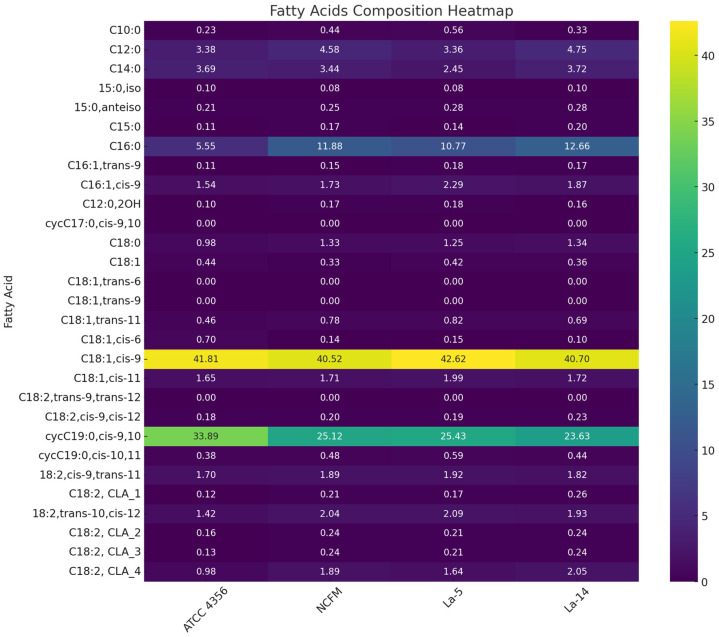
The distribution of the fatty acid composition across the four *Lactobacillus acidophilus* strains.

The fatty acid profile of *L. acidophilus*. The molecular fatty acid profile of the *L. acidophilus* strains ATCC 4356, La-14, La-5, and NCFM was analysed from biomass grown in the medium with the addition of Tween 80^TM^. Twenty-five fatty acids were identified due to the chromatographic separation of fatty acids extracted from the *L. acidophilus* biomass (Table 1, Figure 1). A comparative analysis using Tukey’s test of *L. acidophilus* strains showed no statistically significant differences (*p* < 0.05) in the content of the following fatty acids: C16:1,*cis-*9, C18:1,*cis-*9, C18:2,*cis-*9,*cis-*12, cycC19:0,*cis-*10,11, and branched acids. *L. acidophilus* strains were classified into two groups based on the significant differences in the content of cycC19:0,*cis-*9,10, C16:1,*trans-*9, conjugated 18:2,*cis-*9,*trans-*11, and 18:2,*trans-*10,*cis-*12 acids. The first one includes *L. acidophilus* ATCC 4356, and the second one includes strains La-14, La-5, and NCFM. Two strains of *L. acidophilus*, La-5 and NCFM, were differentiated based on the similar levels of C18:0 and C12:0 acids (*p* < 0.05). A notable feature of the profiles of all these strains is the dominant presence of C18:1,*cis-*9 acid. This may be due to the presence of this acid in the medium. All *L. acidophilus* strains exhibited the ability to synthesise cycC19:0,*cis-*9,10 acid, for which C18:1,*cis-*9 acid was the precursor. C18:1,*cis-*9 acid was predominant in the overall fatty acid profile. C18:1,*cis-*11 and cycC19:0,*cis-*10,11 acids (not exceeding a total of 3% of the overall fatty acid content) were found in low levels, which showed that *L. acidophilus* has a poor ability to synthesise these acids; this may be the reason for the lack of growth of these strains in the medium without Tween 80^TM^. Comparing the proportions of different groups of fatty acids, it was found that the ratio of cycC19:0,*cis-*9,10 acid to cycC19:0,*cis-*10,11 acid was the highest for *L. acidophilus* strains compared to the other strains tested in this study. The ratio of C18:1,*cis-*9 acid to C18:1,*cis-*11 acid was another interesting characteristic of *L. acidophilus* strains, distinguishing them from the *Lactobacillus* strains analysed. Of the other tested *Lactobacillus* strains, only *L. delbrueckii* subsp. *bulgaricus* ATCC11842 showed a higher ratio of C18:1,*cis-*9 acid to C18:1,*cis-*11. In addition, the *L. acidophilus* cells were characterised by a dominating share of conjugated C18:2,*cis-*9,*cis-*12 acids as compared to cells of other *Lactobacillus* species.

**Figure 2:**
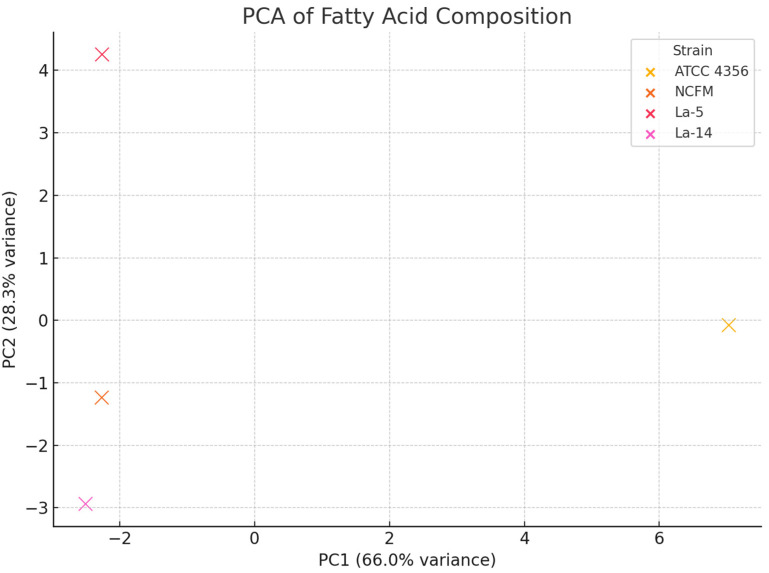
The PCA scatter plot for *Lactobacillus acidophilus* strains. Strains cluster closely, suggesting a general compositional similarity. PC1 explains a significant proportion of variance; the main differences are captured along this axis.

The PCA scatter plot (Figure 2) revealed a clustering pattern that grouped *L. acidophilus* ATCC 4356 separately from La-14, La-5, and NCFM, supporting previous findings regarding their distinct fatty acid signatures, particularly in cycC19:0,*cis-*9,10, C16:1,*trans-*9, and conjugated C18:2 isomers (Table 1). Moreover, ratios of selected fatty acid pairs such as C18:1,*cis-*9/C18:1,*cis-*11 and cycC19:0,*cis-*9,10/cycC19:0,*cis-*10,11 were calculated and found to be the highest in *L. acidophilus* strains, further supporting their unique metabolic phenotype. These findings align with the hypothesis that C18:1,*cis-*9, possibly absorbed from Tween 80^TM^, acts as a precursor for cyclic and conjugated fatty acids in these strains. Taken together, the multivariate and visual analyses contribute complementary evidence to the strain-specific lipidomic profiles, suggesting a possible metabolic grouping and precursor–product relationship within the fatty acid biosynthesis pathway of *L. acidophilus*.

The fatty acid profile of *L. delbrueckii*. The growth of *L. delbrueckii* subsp. *lactis* ATCC 4797 on the MRS agar without the addition of Tween 80^TM^ was challenging and was found to be significantly reduced and slow. Prolonging the incubation period to 48 h did not positively affect the intensity of the bacterial growth. While *L. delbrueckii* subsp. *Lactobacillus bulgaricus* ATCC 11842 showed some growth in the Tween 80-free medium, the resulting biomass was markedly less than that observed for other *Lactobacillus* species used in this study. Twenty fatty acids were identified in the fatty acid profile of the *L. delbrueckii* subsp. *lactis* ATCC 4797 biomass. Twenty-three fatty acids were identified in the composition of the fatty acid profile of *L. delbrueckii* subsp. *bulgaricus* ATCC11842 (Table 2). The molecular fatty acid profile of *L. delbrueckii* subsp. *lactis* ATCC 4797 was dominated by C18:1,*cis-*9 acid. The other fatty acids with a dominant share were C16:0, C18:1 (*cis-*11), and C16:1 (*cis-*9) acids. *L. delbrueckii* subsp. *lactis* ATCC 4797 did not demonstrate the ability to synthesise cyclic acids. The presence of cycC19:0,*cis-*9,10 acid was not observed despite the high content of C18:1,*cis-*9 acid. It can be assumed that the lack of the ability to transform C18:1,*cis-*9 acid or C18:1,*cis-*11 acid into their cyclic derivatives was the cause of the poor growth of this strain in the medium without Tween 80^TM^. Both strains of *L. delbrueckii* showed statistically significant differences in the fatty acid profiles of the cells (*p* < 0.05). The lack of cyclic acids and a dominant share of C18:1,*cis-*9 acid in the profile of the *L. delbrueckii* subsp. *lactis* ATCC 4797 biomass were marked differences. The presence of *cis-*9 and *trans-*9 isomers of C16:0 acid and a significant presence of C18:1,*cis-*11 acid were characteristic of *L. delbrueckii* subsp. *bulgaricus* ATCC11842. This indicates that significant differences in the fatty acid profile of bacterial cells can occur within the same species of *Lactobacillus*. It is worth noting that the comparison of fatty acid shares showed that only *L. delbrueckii* subsp. *bulgaricus* ATCC11842 had the highest ratio of C18:1,*cis-*9 acid to C18:1,*cis-*11 acid. The molecular fatty acid profile of *L. delbrueckii* subsp. *bulgaricus* ATCC11842 and *L. delbrueckii* subsp. *lactis* ATCC 4797 biomass was characterised by a high U/S value and the contribution of saturated fatty acids compared to the fatty acid profile of other *Lactobacillus* strains tested. A significant difference was observed between *L. acidophilus*, *L. delbrueckii* subsp. *bulgaricus* ATCC 11842, *L. delbrueckii* subsp. *lactis* ATCC 4797, and the other tested *Lactobacillus* strains in terms of the synthesis of cycC19:0,*cis-*9,10 and cycC19:0,*cis-*10,11 acids. The strains of *L. acidophilus* demonstrated a weak ability to synthesise cycC19:0,*cis-*10,11 acid despite the presence of substrates for its formation (C18:1,*cis-*11 acid), and *L. delbrueckii* subsp. *lactis* ATCC 4797 was the only strain that demonstrated an inability to synthesise cyclopropane acids. However, this did not prevent growth entirely in the medium without Tween 80™, although only minimal visible growth was attained. An increased share of cycC19:0,*cis-*9,10 and C18:1,*cis-*9 acids in the bacterial cells cultured in the medium containing Tween 80^TM^ was observed in each case, except for *L. delbrueckii* subsp. *lactis* ATCC 4797.

The fatty acid profile of *L. helveticus*. *L. helveticus* LH-B01 is a thermophilic strain commonly used in commercial cheese production but not in the production of fermented milk, like the previously discussed strains. This strain demonstrates good growth in the medium with and without Tween 80^TM^. Its fatty acid profile was characterised by the presence of twenty-seven fatty acids, both in the medium with and without Tween 80^TM^. C16:0, C18:1,*cis-*11, and cycC19:0,*cis-*10,11 acids were found at levels greater than 15% in the profile obtained in the medium without Tween 80^TM^. On the other hand, dominant levels of C16:0 and C18:1,*cis-*9 acids were identified in the profile obtained in the medium with the addition of Tween 80^TM^. Both cyclic acids have a significant share in the fatty acid profile obtained from the medium supplemented with Tween 80^TM^, in contrast to the fatty acid profile obtained from the medium without Tween 80^TM^, in which the share of cycC19:0,*cis-*9,10 acid was 1.78% (Table 2).

**Figure 3:**
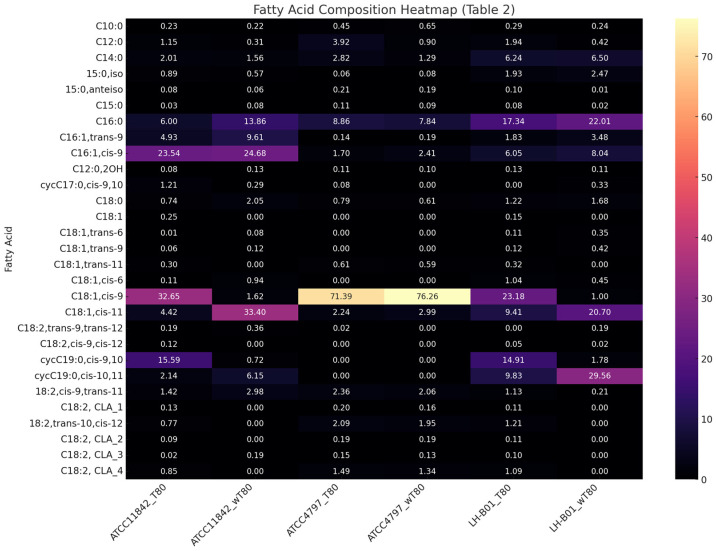
The distribution of fatty acids of *Lactobacillus* strains (*L. delbrueckii* subsp. bu*l*garicus ATCC11842, *L. delbrueckii* subsp. *lactis* ATCC4797, and *L. helveticus* LH-B01) grown with and without Tween 80^TM^ (T80/wT80). Clear differences were observed between the fatty acid profiles of strains cultured with (T80) and without (wT80) Tween 80^TM^. The absence of Tween 80^TM^ led to a marked reduction in C18:1,*cis-*9, as well as CLA isomers and cyclic fatty acids, confirming Tween 80^TM^ as a key contributor or precursor to their synthesis. Conversely, levels of C16:0 and C18:1,*cis-*11 increased under wT80 conditions in some strains, suggesting a compensatory adjustment in the membrane lipid composition to maintain functional integrity in the absence of exogenous unsaturated fatty acids.

**Figure 4:**
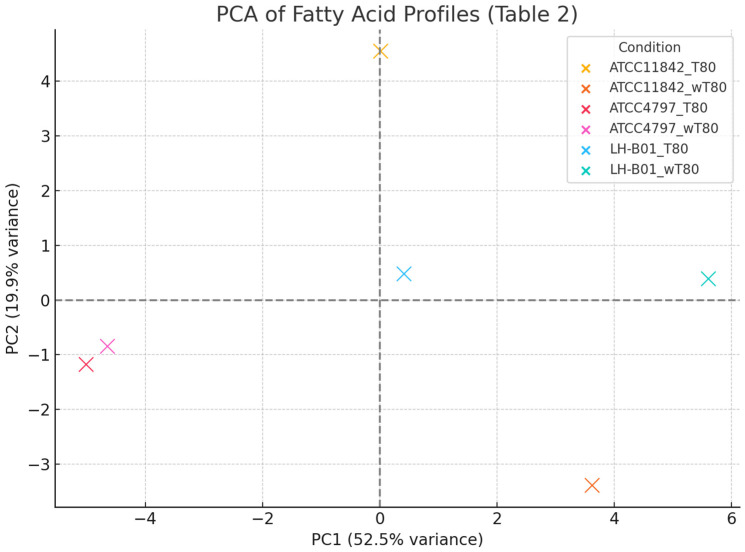
The PCA scatter plot for *Lactobacillus* strains (*L. delbrueckii* subsp. *bulgaricus* ATCC11842, *L. delbrueckii* subsp. *lactis* ATCC4797, and *L. helveticus* LH-B01) grown with and without Tween 80^TM^ (T80/wT80). The PCA revealed a clear separation between strains and treatment groups, with PC1 accounting for the majority of the variance, primarily driven by the presence or absence of Tween 80^TM^. Notably, the LH-B01_wT80 profile was highly distinct, occupying the lower-right quadrant, indicating a unique fatty acid composition under this condition.

To better understand the metabolic divergence among the *L. delbrueckii* strains and *L. helveticus* LH-B01 under different culture conditions, a multivariate statistical analysis was conducted based on the fatty acid composition from Table 2. A heatmap visualisation (Figure 3) of the fatty acid content across all conditions further confirmed major differences between strains and treatments. *L. delbrueckii* subsp. *lactis* ATCC 4797 showed an almost complete absence of cyclic fatty acids under both conditions, despite high C18:1,*cis-*9 contents. This supports the hypothesis that the strain lacks the enzymatic machinery to convert oleic acid into cyclic derivatives, which may explain its limited growth in media without Tween 80^TM^. A Principal Component Analysis (Figure 4) demonstrated distinct clustering between strains and culture conditions, with PC1 accounting for the majority of the variance. Notably, *L. helveticus* LH-B01 grown without Tween 80™ displayed the most distinct separation, likely due to a shift toward higher C16:0 and C18:1,*cis-*11 contents and a sharp reduction in conjugated and cyclic fatty acids, such as cycC19:0,*cis-*9,10 and CLA isomers. As shown in Table 2, a marked increase in palmitic acid (C16:0) was observed in the L. helveticus LH-B01 cultured without Tween 80™, indicating a potential shift toward saturated fatty acid synthesis in the absence of external oleic acid precursors. This could reflect a compensatory mechanism that maintains membrane fluidity under the stress conditions associated with the deprivation of Tween 80^TM^. Across all strains, the level of oleic acid (C18:1,*cis-*9) decreased significantly when Tween 80™ was removed from the medium. This strongly suggests that Tween 80^TM^ is either a direct source of C18:1,*cis-*9 or plays an essential role in its biosynthesis or uptake. Given the known composition of Tween 80^TM^, this finding is consistent with its contribution to the pool of unsaturated fatty acids in bacterial membranes. In the *L. helveticus* LH-B01 grown without Tween 80™, the level of the cyclic fatty acid cycC19:0,*cis-*10,11 rose sharply. This unusual increase may reflect a strain-specific adaptation, where the cyclopropanation of unsaturated fatty acids is upregulated in response to membrane rigidity or environmental stress. Notably, this response was not observed in other strains, highlighting the unique metabolic capability of LH-B01. *L. delbrueckii* subsp. *bulgaricus* ATCC11842 exhibited the highest ratio of C18:1,*cis-*9 to C18:1,*cis-*11, indicating a preferential accumulation or utilisation of oleic acid. This lipid profile may be associated with improved membrane fluidity or stability and could be a distinguishing feature of this subspecies. The dominance of C18:1,*cis-*9 also correlates with this strain’s better growth performance in the absence of Tween 80^TM^, compared to *L. delbrueckii* subsp. *lactis*.

Although the metabolic effects of Tween 80 were evaluated in this study, we did not perform a comparative growth curve analysis of the strains in the presence and absence of Tween 80. This additional experiment could provide complementary insights into whether Tween 80 influences growth kinetics, toxicity, or substrate utilisation.

## 3. Discussion

The results of this study clearly demonstrate that the fatty acid profiles of thermophilic *Lactobacillus* strains are substantially influenced by the presence or absence of Tween 80^TM^, a non-ionic surfactant and oleic acid donor. The strain-dependent ability to grow and synthesise specific fatty acids in media devoid of Tween 80^TM^ provides both physiological and chemotaxonomic insight into their lipid metabolism. Notably, *L. acidophilus* strains failed to grow without Tween 80™, which aligns with the hypothesis that these strains are incapable of synthesising essential fatty acids such as oleic acid (C18:1,*cis-*9), a finding previously reported by Partenen et al. [2] and Johnsson et al. [4].

**Figure 5:**
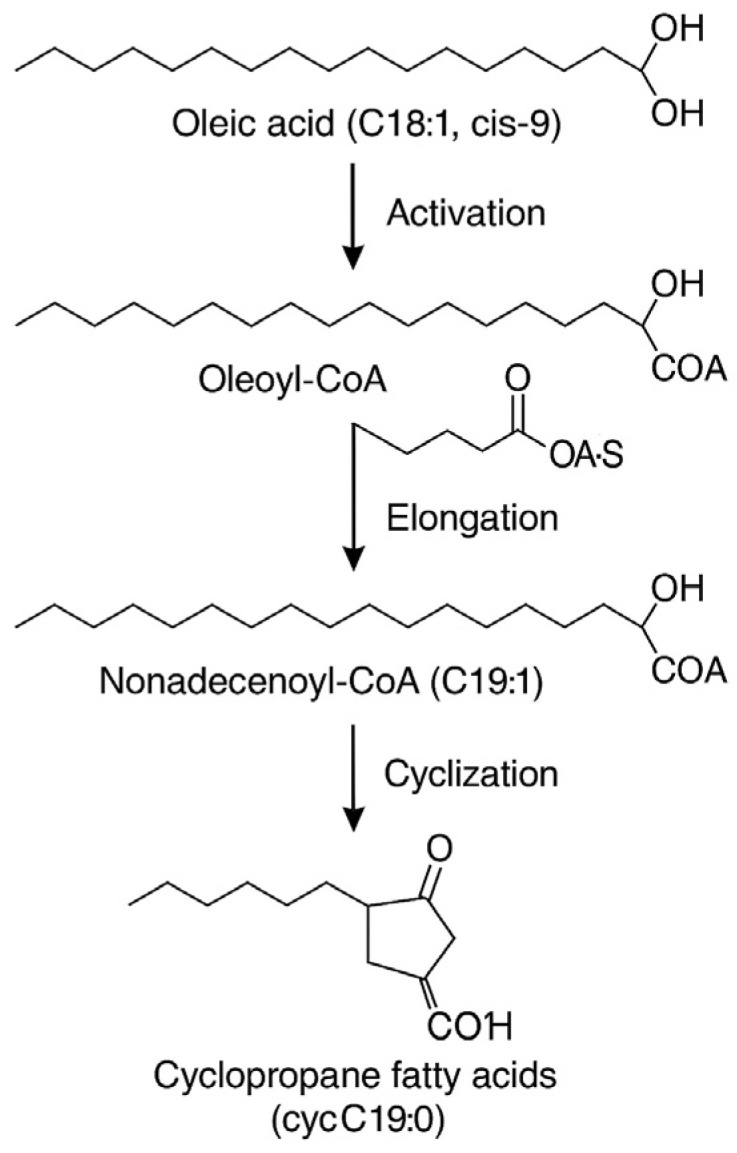
Conversion of oleic acid (C18:1,*cis-*9) to cyclic fatty acids (cycC19:0). Source: authors’ material.

The uptake and bioconversion of unsaturated fatty acids into cyclic fatty acids (Figure 5), such as lactobacillic acid (cycC19:0,*cis-*10,11) and dihydrosterculic acid (cycC19:0,*cis-*9,10), was confirmed across most strains in the presence of Tween 80^TM^, suggesting active enzymatic transformation mechanisms. Although our results provide strong indirect evidence of Tween 80 uptake and oleic acid conversion, direct confirmation (e.g., via isotopically labelled substrates) was beyond the scope of this study and should be addressed in future research. This cyclopropanation is presumed to enhance membrane rigidity, aiding bacterial survival under stress conditions [3,9]. This mechanism involves the activation of oleic acid into oleoyl-CoA, followed by its conversion via cyclopropane fatty acid synthase at the Δ9 or Δ11 double bond position [3,9].

Despite Tween 80^TM^ primarily being a source of oleic acid, the expected elevation in C18:1,*cis-*9 was not consistent across strains [10,11]. Interestingly, in our study, not all strains responded uniformly. For example, *L. delbrueckii* subsp. lactis ATCC 4797 showed high levels of C18:1,*cis-*9, but lacked any detectable cyclic fatty acids. This absence strongly indicates an enzymatic deficiency in cyclopropanation pathways. This metabolic difference may underlie the poor growth observed (or significantly reduced growth) in Tween 80™-free media, as compared to strains capable of cyclopropanation.

The multivariate analyses, including the Principal Component Analysis (PCA) and heatmaps, provided an additional layer of evidence for the metabolic divergence across strains. Particularly, *L. acidophilus* ATCC 4356 was clearly separated from La-14, La-5, and NCFM via higher levels of cycC19:0,*cis-*9,10 and specific CLA isomers. These distinctions confirm the strain-specific nature of lipid biosynthesis and support previous findings suggesting that *L. acidophilus* strains possess isomerase activity for CLA synthesis [12,13,14,15,16,17,18,19].

Notably, the ratios of C18:1,*cis-*9 to C18:1,*cis-*11 and cycC19:0,*cis-*9,10 to cycC19:0,*cis-*10,11 emerged as discriminative molecular markers, capable of differentiating strains within and between species. These ratios may thus serve as chemotaxonomic indicators, which is consistent with the findings of Partanen et al. [2] and Macouzet et al. [13]. However, these findings also underscore the analytical challenges associated with resolving structurally similar isomers. The overlapping mass spectra of positional isomers (e.g., *cis-*9 vs. *cis-*11) or cyclic derivatives necessitate highly specific GC-MS methods, as evidenced by discrepancies between our findings and those of Rizzo et al. [20].

The comparison with previous studies revealed several methodological and biological differences. For instance, for *L. acidophilus* ATCC 4356 Liong and Shah [12] reported a fatty acid profile with a notably higher proportion of saturated fatty acids and C18:0 than what we observed in our study. One plausible explanation is that cyclic fatty acids were not included in their analysis, significantly skewing the saturated/unsaturated acid ratio. Furthermore, methodological limitations in their analytical system may have led to the underreporting of conjugated or cyclic compounds, especially those derived from C18:1,*cis-*9.

Our observations for *L. helveticus* LH-B01 suggest a robust capacity for cyclopropane fatty acid synthesis, even with Tween 80^TM^ deprivation, particularly for cycC19:0,*cis-*10,11. This contrasts with *L. delbrueckii* subsp. *lactis*, reinforcing the idea that metabolic capabilities are strain-specific and influenced by both genetic factors and culture conditions. Such adaptation mechanisms are likely regulated through membrane lipid remodelling pathways that favour the synthesis of saturated or cyclic fatty acids to maintain membrane integrity under stress [1,21,22].

The presence of multiple CLA isomers, particularly in *L. acidophilus* and *L. helveticus*, is consistent with previously proposed pathways involving linoleic acid isomerase and desaturase activity [17,19,23,24]. These enzymatic activities convert C18:1,*cis-*9 into C18:2,*cis-*9,*cis-*12 and into CLA isomers such as 18:2,*cis-*9,*trans-*11 and 18:2,*trans-*10,*cis-*12, both of which were found in varying proportions across strains. This biosynthetic potential, coupled with the influence of Tween 80^TM^, may inform future optimism regarding culture conditions for the enhanced production of functional lipids.

In summary, this study reaffirms the critical influence of extracellular oleic acid (such as Tween 80^TM^) on the lipidomic architecture of thermophilic *Lactobacillus* strains. Despite Tween 80^TM^ primarily being a source of oleic acid, its composite nature, containing minor fatty acids, introduces complexity in interpreting its exact metabolic role [3,4,7]. These nuances, combined with strain-specific enzymatic capacities, shape the observed diversity in fatty acid profiles. The integration of molecular, chromatographic, and multivariate approaches has provided comprehensive insight into membrane lipid remodelling in lactic acid bacteria, revealing biomarkers with potential chemotaxonomic and biotechnological relevance.

A limitation of this study is the lack of a comparative growth profile of the strains in the presence and absence of Tween 80. Such an analysis would provide additional context regarding whether Tween 80 exerts any growth-promoting or inhibitory effects or if it serves as a potential carbon source. Future studies should address this by including detailed growth kinetics to complement the current metabolic and biochemical data. Although our results strongly suggest the uptake of oleic acid from Tween 80, a direct confirmation (e.g., via isotopically labelled oleic acid) was beyond the scope of this study and should be pursued in future research.

## 4. Materials and Methods

### 4.1. Materials

This study was based on seven certified strains: *L. acidophilus* ATCC 4356, *L. acidophilus* La-14, *L. acidophilus* La-5, *L. acidophilus* NCFM, *L. delbrueckii* subsp. *bulgaricus* ATCC 11842, *L. delbrueckii* subsp. *lactis* ATCC 4797, and *L. helveticus* LH-B01. Each strain was cultured in de Man–Rogosa–Sharpe (MRS) agar (Merck, Darmstadt, Germany) with or without 0.1% (*v*/*v*) Tween 80^TM^. The MRS composition per litre was as follows: peptone 10 g, beef extract 8 g, yeast extract 4 g, glucose 20 g, dipotassium phosphate 2 g, sodium acetate 5 g, tri-ammonium citrate 2 g, magnesium sulphate 0.2 g, and manganese sulphate 0.05 g. Cultures were incubated at 37 °C for 24 h under microaerophilic conditions. The biomass of the obtained monocultures was used to extract fatty acids. Biomass was harvested by centrifugation at 12,000× *g* for 10 min at 6 °C and washed twice with sterile saline. A total of six replicates for each strain and type of medium were performed.

### 4.2. Extraction of Fatty Acids from Bacterial Biomass

Fatty acid extraction and methylation were conducted according to a modified alkaline–acidic transesterification method [7,23,24]. Approximately 50 mg of bacterial biomass was suspended in 2 mL of a methanolic NaOH solution (15% *w*/*v*) and incubated at 100 °C for 5 min to achieve saponification. After cooling, 2 mL of a methylation reagent (14% BF_3_ in methanol) was added, and the mixture was heated again at 100 °C for 10 min to convert the liberated fatty acids into their methyl esters (FAMEs). The reaction was stopped by adding 1 mL of deionised water, and FAMEs were extracted twice with 2 mL of n-hexane. The combined organic layers were dried over anhydrous sodium sulphate, filtered, and evaporated under a gentle nitrogen stream. The dry residue was redissolved in 100 µL of n-hexane and stored at −20 °C until GC-MS analysis. This method is characterised by the acidic extraction of fatty acids, which enables effective penetration of the LAB cell wall and extraction of fatty acids characteristic of bacterial biomass, such as branched, cyclic, hydroxy, and unsaturated fatty acids. Moreover, the saponification step enables the extraction and subsequent methylation of all fatty acids, including those that are unesterified. However, the acid methylation method was used due to its effective extraction of unstable fatty acids such as hydroxy and cyclopropane fatty acids [7,20,24,25,26,27,28,29].

### 4.3. Conditions for Separating and Detecting Fatty Acid Methyl Esters Using GC-MS

Chromatographic separation of fatty acid methyl esters was carried out by gas chromatography coupled with a mass spectrometer (GC-MS QP2010, Shimadzu Corporation, Kyoto, Japan) in a polar column (30 m × 0.25 mm × 0.20 μm; Quadrex Corporation, Woodbridge, CT, USA). Helium was used as the carrier gas at a flow rate of 1 mL/min. The injector was maintained at 250 °C in split mode (split ratio 1:20). The oven temperature was programmed as follows: initial 60 °C (2 min), ramped to 180 °C at 10 °C/min, then to 230 °C at 3 °C/min, and held for 10 min. The MS detector operated in EI (Electron Ionisation) mode (70 eV), scanning from *m*/*z* 50 to 500, with an ion source temperature of 200 °C. Increased sensitivity of the column and more efficient separation of fatty acids were achieved by using a column with a reduced stationary phase thickness of 0.2 μm, as confirmed by Basconcillo and McCarry [25]. Fatty acid methyl esters were identified by comparing the obtained mass spectra, retention times, and chain length equivalents with external standards of analysed fatty acids and the WILEY7N2 and NIST147 spectral libraries [30]. The standards of C18:1,*cis-*9, C15:0,anteiso, and BAME acids mix (Sigma-Aldrich, Milwaukee, WI, USA); GLC-674 and GLC-617 standards (Nu-Chek-Prep., Elysian, MN, USA); and the mixture of isomers of methyl esters of C18:2,*cis-*9,*cis-*12 acid, 18:2 *cis-*9,*trans-*11, and 18:2 *trans-*10,*cis-*12 (Nu-Chek-Prep., USA) were used to identify the compounds. Primary identification of the cyclic fatty acids was established by authentic standards from the Bacterial Acid Methyl Esters (BAMEs; Table 3) mix, with concordant retention time/equivalent chain length (ECL) and diagnostic EI fragments (e.g., *m*/*z* 69, 74, 83, 97, 123, 278) matching the standards; literature [1,31] was used only for secondary confirmation. Conjugated C18:2 isomers (CLA) were identified using an authentic CLA isomer mixture (Nu-Chek) with agreement in RT/ECL and characteristic EI fragments, alongside WILEY/NIST library matches (Table 4). The nomenclature of fatty acids was compared with the literature data [32]. Chromatographic data were analysed using GC-MS Solution v.2.50 software. Averages and standard deviations were calculated using Microsoft Excel 2010. Statistical analysis was performed using Statistica v.10 software.

### 4.4. Statistical Analysis

Fatty acid composition data were analysed using multivariate and univariate statistical methods to evaluate differences between bacterial strains and culture conditions. The one-way analysis of variance (ANOVA) followed by Tukey’s Honest Significant Difference (HSD) test was applied to identify statistically significant differences between groups (*p* < 0.05). Data visualisation included Principal Component Analysis (PCA) to identify sample groupings and key contributors to variance in the dataset, as well as heatmaps to illustrate overall distribution patterns. All statistically significant differences are reported in Table 1 and Table 2. All analyses were conducted using Python (v3.10) with the following libraries: pandas, scipy, statsmodels, matplotlib, seaborn, and scikit-learn.

## 5. Conclusions

The present study provides comprehensive molecular insights into the fatty acid (FA) profiles of thermophilic *Lactobacillus* strains, highlighting the critical role of Tween 80^TM^ as a source of exogenous oleic acid. Utilising advanced chromatographic and spectrometric techniques, we demonstrated that the addition of Tween 80^TM^ significantly alters the lipidomic architecture of bacterial membranes. These modifications include a marked increase in cyclic fatty acids, such as lactobacillic (cycC19:0,*cis-*10,11) and dihydrosterculic acids (cycC19:0,*cis-*9,10), and shifts in the proportions of unsaturated and conjugated fatty acids, such as CLA isomers. The strain-specific variability in the uptake and transformation of C18:1,*cis-*9 acid confirms the complexity of the metabolic responses within thermophilic *Lactobacillus* strains [3,4,9].

One of the key findings of this study is the identification of molecular markers, namely the C18:1,*cis-*9/C18:1,*cis-*11 and cycC19:0,*cis-*9,10/cycC19:0,*cis-*10,11 ratios, that offer high-resolution discrimination between strains and can serve as potential chemotaxonomic indicators [2,3,11]. These ratios may reflect specific enzymatic capacities for fatty acid cyclopropanation and desaturation, thereby enhancing our understanding of lipid metabolism and adaptive physiology in lactic acid bacteria. The observed elevation of cyclic fatty acids in strains cultured with Tween 80^TM^ supports the hypothesis of a strain-dependent membrane remodelling mechanism in response to external oleic acid [3,9].

Importantly, we also identified analytical limitations related to the chromatographic separation of structurally similar isomers, particularly in distinguishing between C18:1,*cis-*9 and C18:1,*cis-*11, as well as between cycC19:0 positional isomers. This limitation underscores the necessity for further methodological refinement to ensure an accurate structural resolution and quantification of lipid species [3,20]. A limitation of this study is the lack of direct tracer experiments confirming Tween 80 uptake; therefore, our conclusions are based on indirect chromatographic evidence. Future work should also include comparative growth analyses to clarify the physiological impact of Tween 80 on these strains.

Overall, this study enhances our understanding of the molecular lipid composition of *Lactobacillus* membranes and underscores the influence of the medium composition on microbial physiology. These findings not only offer a foundation for chemotaxonomic classification and strain differentiation but also support the rational design of probiotic strains with tailored fatty acid profiles for industrial and functional applications [11,13,20].

## Figures and Tables

**Table 1:** The fatty acid composition (%) of *Lactobacillus acidophilus* strains obtained from the cells cultured in a medium with Tween 80^TM^.

Fatty Acid (Acid Name)/Strain Symbol	Class	Precursor of cyc/CLA	ATCC 4356	NCFM	La-5	La-14
C10:0 (caproic/decanoic)	saturated	–	0.23 ^a^ ± 0.01	0.44 ^b^ ± 0.10	0.56 ^b^ ± 0.24	0.33 ^a,b^ ± 0.13
C12:0 (lauric/dodecanoic)	saturated	–	3.38 ^a^ ± 0.34	4.58 ^b^ ± 0.55	3.36 ^a^ ± 2.52	4.75 ^b^ ± 0.54
C14:0 (myristic/tetradecanoic)	saturated	–	3.69 ^a^ ± 0.19	3.44 ^a^ ± 0.30	2.45 ^b^ ± 1.03	3.72 ^a^ ± 0.31
15:0,iso (iso-13-methyltetradecanoic)	saturated (branched)	–	0.10 ^a^ ± 0.02	0.08 ^a^ ± 0.02	0.08 ^a^ ± 0.01	0.10 ^a^ ± 0.02
15:0,anteiso (anteiso-12-methyltetradecanoic)	saturated (branched)	–	0.21 ^a^ ± 0.02	0.25 ^a,b^ ± 0.03	0.28 ^b^ ± 0.03	0.28 ^b^ ± 0.04
C15:0 (pentadecanoic) C16:0 (palmitic/hexadecanoic)	saturated	–	0.11 ^a^ ± 0.02	0.17 ^b^ ± 0.02	0.14 ^b^ ± 0.04	0.20 ^b^ ± 0.05
	saturated	–	5.55 ^a^ ± 0.35	11.88 ^b^ ± 0.72	10.77 ^b^ ± 1.53	12.66 ^b^ ± 1.13
C16:1,*trans-*9 (palmitelaidic/*trans-*9-hexadecenoic) C16:1,*cis-*9 (palmitoleic/*cis-*9-hexadecenoic)	unsaturated (trans)	–	0.11 ^a^ ± 0.01	0.15 ^a,b^ ± 0.02	0.18 ^b^ ± 0.02	0.17 ^b^ ± 0.03
	unsaturated (cis)	–	1.54 ^a^ ± 0.09	1.73 ^a,b^ ± 0.18	2.29 ^b^ ± 0.54	1.87 ^a,b^ ± 0.20
C12:0,2OH (2-hydroxydodecanoic)	saturated hydroxyacid	–	0.10 ^a^ ± 0.02	0.17 ^a^ ± 0.02	0.18 ^a^ ± 0.06	0.16 ^a^ ± 0.03
cycC17:0,*cis-*9,10 (*cis-*9,10-methylenehexadecanoic)	cyclic	–	0.00 ^a^ ± 0.00	0.00 ^a^ ± 0.00	0.00 ^a^ ± 0.00	0.00 ^a^ ± 0.00
C18:0 (stearic/octadecanoic)	saturated	–	0.98 ^a^ ± 0.11	1.33 ^b^ ± 0.17	1.25 ^b^ ± 0.16	1.34 ^b^ ± 0.30
C18:1 (octadecenoic)	unsaturated	–	0.44 ^b^ ± 0.08	0.33 ^a^ ± 0.07	0.42 ^b^ ± 0.02	0.36 ^a^ ± 0.05
C18:1,*trans-*6 (petroselaidic/*trans-*6-octadecenoic)	unsaturated (trans)	–	0.00 ^a^ ± 0.00	0.00 ^a^ ± 0.00	0.00 ^a^ ± 0.00	0.00 ^a^ ± 0.00
C18:1,*trans-*9 (elaidic/*trans-*9-octa-decenoic)	unsaturated (trans)	–	0.00 ^a^ ± 0.00	0.00 ^a^ ± 0.00	0.00 ^a^ ± 0.00	0.00 ^a^ ± 0.00
C18:1,*trans-*11 (*trans-*vaccenic/*trans-*11-octadecenoic)	unsaturated (trans)	–	0.46 ^a^ ± 0.04	0.78 ^b^ ± 0.15	0.82 ^b^ ± 0.06	0.69 ^b^ ± 0.31
C18:1,*cis-*6 (petroselinic/*cis-*6-octadecenoic)	unsaturated (cis)	–	0.70 ^b^ ± 0.06	0.14 ^a^ ± 0.05	0.15 ^a^ ± 0.03	0.10 ^a^ ± 0.02
C18:1,*cis-*9 (oleic/*cis-*9-octadecenoic)	unsaturated (cis)	* (CLA and cyclic acid precursor)	41.81 ^a^ ± 0.79	40.52 ^a^ ± 3.11	42.62 ^a^ ± 1.11	40.70 ^a^ ± 2.11
C18:1,*cis-*11 (*cis-*vaccenic/*cis-*11-octadecenoic)	unsaturated (cis)	–	1.65 ^a^ ± 0.02	1.71 ^a^ ± 0.06	1.99 ^b^ ± 0.28	1.72 ^a^ ± 0.02
C18:2,*trans-*9,*trans-*12 (linoelaidic/*trans-*9, *trans-*12-octadecadienoic)	unsaturated (trans)	–	0.00 ^a^ ± 0.00	0.00 ^a^ ± 0.00	0.00 ^a^ ± 0.00	0.00 ^a^ ± 0.00
C18:2,*cis-*9,*cis-*12 (linoleic/*cis-*9,*cis* 12-octadecadienoic)	unsaturated (cis)	* (CLA precursor)	0.18 ^a^ ± 0.02	0.20 ^a^ ± 0.03	0.19 ^a^ ± 0.07	0.23 ^a^ ± 0.07
cycC19:0,*cis-*9,10 (dihydrosterculic/*cis-*9,10-methyleneoctadecanoic)	cyclic	–	33.89 ^a^ ± 0.84	25.12 ^b^ ± 3.01	25.43 ^b^ ± 5.45	23.63 ^b^ ± 2.55
cycC19:0,*cis-*10,11 (lactobacillic/*cis-*11,12-methyleneoctadecanoic)	cyclic	–	0.38 ^a^ ± 0.26	0.48 ^a^ ± 0.05	0.59 ^a^ ± 0.10	0.44 ^a^ ± 0.06
18:2,*cis-*9,*trans-*11 (conjugated octadecadienoic)	conjugated (CLA)	–	1.70 ^a^ ± 0.05	1.89 ^a^ ± 0.22	1.92 ^b^ ± 0.10	1.82 ^a^ ± 0.24
C18:2, CLA_1 (conjugated octadecadienoic)	conjugated (CLA)	–	0.12 ^a^ ± 0.02	0.21 ^a^ ± 0.05	0.17 ^a^ ± 0.03	0.26 ^b^ ± 0.06
18:2,*trans-*10,*cis-*12 (conjugated octadecadienoic)	conjugated (CLA)	–	1.42 ^a^ ± 0.04	2.04 ^a,b^ ± 0.22	2.09 ^b^ ± 0.12	1.93 ^a,b^ ± 0.25
C18:2, CLA_2 (conjugated octadecadienoic)	conjugated (CLA)	–	0.16 ^a^ ± 0.02	0.24 ^b^ ± 0.04	0.21 ^b^ ± 0.04	0.24 ^b^ ± 0.02
C18:2, CLA_3 (conjugated octadecadienoic)	conjugated (CLA)	–	0.13 ^a^ ± 0.01	0.24 ^b^ ± 0.02	0.21 ^b^ ± 0.03	0.24 ^b^ ± 0.02
C18:2, CLA_4 (conjugated octadecadienoic)	conjugated (CLA)	–	0.98 ^a^ ± 0.02	1.89 ^a,b^ ± 0.23	1.64 ^a,b^ ± 0.31	2.05 ^b^ ± 0.18
ratio cycC19:0,*cis-*9,10/cycC19:0,*cis-*10,11			89.18	52.33	43.10	53.70
ratio C18:1,*cis-*9/C18:1,*cis-*11			25.34	23.70	21.42	23.66
∑ unsaturated acids			51.40	52.07	54.90	52.38
∑ saturated acids			14.25	22.17	18.89	23.38
ratio unsaturated/saturated			3.61	2.35	2.91	2.24
∑ conjugated C18:2,*cis-*9,*cis-*12 acids			4.51	6.51	6.24	6.54
cis/trans			47.37	44.10	46.85	44.53
mono-/polyunsaturated			5.89	4.83	6.50	5.83
CLA/other conjugated fatty acids			6.87	7.44	7.72	8.97

Legend: Values are presented as mean ± standard deviation (n = 6). ^a,b^ Different superscript letters within the same row indicate statistically significant differences (*p* < 0.05) between *L. acidophilus* strains according to one-way ANOVA and Tukey’s post hoc test. Asterisks (*) denote fatty acids that are precursors of cyclic or conjugated linoleic acids (CLAs).

**Table 2:** The fatty acid composition (%) of *Lactobacillus* strains obtained from the cells cultured in a medium with Tween 80^TM^ (T80) and without Tween 80^TM^ (wT80).

Strain Symbol	*L. delbrueckii* subsp. *bulgaricus* ATCC 11842		*L. delbrueckii* subsp. *lactis* ATCC 4797		*L. helveticus* LH-B01	
Fatty Acid/Medium	T80	wT80	T80	wT80	T80	wT80
C10:0 (caproic/decanoic)	0.23 ^a^ ± 0.07	0.22 ^a^ ± 0.02	0.45 ^a,b^ ± 0.21	0.65 ^b^ ± 0.20	0.29 ^a^ ± 0.05	0.24 ^a^ ± 0.15
C12:0 (lauric/dodecanoic)	1.15 ^a^ ± 0.58	0.31 ^b^ ± 0.06	3.92 ^c^ ± 1.51	0.90 ^b^ ± 0.13	1.94 ^a,b^ ± 0.20	0.42 ^b^ ± 0.17
C14:0 (myristic/tetradecanoic)	2.01 ^a^ ± 0.24	1.56 ^a^ ± 0.43	2.82 ^a^ ± 0.65	1.29 ^b^ ± 0.13	6.24 ^c^ ± 1.93	6.50 ^c^ ± 2.60
15:0,iso (iso-13-methyltetradecanoic)	0.89 ^a^ ± 0.15	0.57 ^b^ ± 0.07	0.06 ^c^ ± 0.00	0.08 ^c^ ± 0.03	1.93 ^d^ ± 0.55	2.47 ^d^ ± 0.71
15:0,anteiso (anteiso-12-methyltetradecanoic)	0.08 ^a^ ± 0.07	0.06 ^a^ ± 0.03	0.21 ^b^ ± 0.02	0.19 ^b^ ± 0.01	0.10 ^a^ ± 0.01	0.01 ^c^ ± 0.01
C15:0 (pentadecanoic) C16:0 (palmitic/hexadecanoic)	0.03 ^a^ ± 0.05	0.08 ^a^ ± 0.05	0.11 ^b^ ± 0.01	0.09 ^b^ ± 0.02	0.08 ^a,b^ ± 0.04	0.02 ^a^ ± 0.03
	6.00 ^a^ ± 0.87	13.86 ^b^ ± 0.67	8.86 ^c^ ± 0.49	7.84 ^c^ ± 0.48	17.34 ^d^ ± 1.24	22.01 ^e^ ± 2.78
C16:1,*trans-*9 (palmitelaidic/*trans-*9-hexadecenoic) C16:1,*cis-*9 (palmitoleic/*cis-*9-hexadecenoic)	4.93 ^a^ ± 1.26	9.61 ^b^ ± 0.30	0.14 ^c^ ± 0.03	0.19 ^c^ ± 0.04	1.83 ^d^ ± 0.84	3.48 ^d^ ± 1.05
	23.54 ^a^ ± 4.80	24.68 ^a^ ± 1.35	1.70 ^b^ ± 0.44	2.41 ^b^ ± 0.02	6.05 ^c^ ± 1.22	8.04 ^c^ ± 2.28
C12:0,2OH (2-hydroxydodecanoic)	0.08 ^a^ ± 0.07	0.13 ^a^ ± 0.04	0.11 ^a^ ± 0.03	0.10 ^a^ ± 0.02	0.13 ^a^ ± 0.01	0.11 ^a^ ± 0.04
cycC17:0,*cis-*9,10 (*cis-*9,10-methylenehexadecanoic)	1.21 ^a^ ± 0.41	0.29 ^b^ ± 0.05	0.08 ^b^ ± 0.13	0.00 ^b^ ± 0.00	0.00 ^b^ ± 0.00	0.33 ^b^ ± 0.11
C18:0 (stearic/octadecanoic)	0.74 ^a^ ± 0.07	2.05 ^b^ ± 0.04	0.79 ^a^ ± 0.10	0.61 ^a^ ± 0.06	1.22 ^c^ ± 0.22	1.68 ^c^ ± 0.87
C18:1 (octadecenoic)	0.25 ^a^ ± 0.06	0.00 ^b^ ± 0.00	0.00 ^b^ ± 0.00	0.00 ^b^ ± 0.00	0.15 ^a^ ± 0.02	0.00 ^b^ ± 0.00
C18:1,*trans-*6 (petroselaidic/*trans-*6-octadecenoic)	0.01 ^a^ ± 0.02	0.08 ^a^ ± 0.02	0.00 ^a^ ± 0.00	0.00 ^a^ ± 0.00	0.11 ^b^ ± 0.04	0.35 ^c^ ± 0.07
C18:1,*trans-*9 (elaidic/*trans-*9-octa-decenoic)	0.06 ^a^ ± 0.03	0.12 ^a^ ± 0.02	0.00 ^b^ ± 0.00	0.00 ^b^ ± 0.00	0.12 ^a^ ± 0.07	0.42 ^c^ ± 0.06
C18:1,*trans-*11 (*trans-*vaccenic/*trans-*11-octadecenoic)	0.30 ^a^ ± 0.07	0.00 ^b^ ± 0.00	0.61 ^c^ ± 0.19	0.59 ^c^ ± 0.26	0.32 ^a^ ± 0.07	0.00 ^b^ ± 0.00
C18:1,*cis-*6 (petroselinic/*cis-*6-octadecenoic)	0.11 ^a^ ± 0.06	0.94 ^b^ ± 0.09	0.00 ^c^ ± 0.00	0.00 ^c^ ± 0.00	1.04 ^b^ ± 0.30	0.45 ^a,b^ ± 0.34
C18:1,*cis-*9 (oleic/*cis-*9-octadecenoic)	32.65 ^a^ ± 4.99	1.62 ^b^ ± 0.70	71.39 ^c^ ± 2.67	76.26 ^c^ ± 1.33	23.18 ^d^ ± 2.22	1.00 ^b^ ± 0.21
C18:1,*cis-*11 (*cis-*vaccenic/*cis-*11-octadecenoic)	4.42 ^a^ ± 0.68	33.40 ^b^ ± 2.22	2.24 ^c^ ± 0.31	2.99 ^c^ ± 0.13	9.41 ^d^ ± 1.84	20.70 ^e^ ± 7.19
C18:2,*trans-*9,*trans-*12 (linoelaidic/*trans-*9, *trans-*12-octadecadienoic)	0.19 ^a^ ± 0.03	0.36 ^a^ ± 0.06	0.02 ^b^ ± 0.04	0.00 ^b^ ± 0.00	0.00 ^b^ ± 0.00	0.19 ^a^ ± 0.10
C18:2,*cis-*9,*cis-*12 (linoleic/*cis-*9,*cis* 12-octadecadienoic)	0.12 ^a^ ± 0.06	0.00 ^b^ ± 0.00	0.00 ^b^ ± 0.00	0.00 ^b^ ± 0.00	0.05 ^a,b^ ± 0.05	0.02 ^a,b^ ± 0.03
cycC19:0,*cis-*9,10 (dihydrosterculic/*cis-*9,10-methyleneoctadecanoic)	15.59 ^a^ ± 2.33	0.72 ^b^ ± 0.37	0.00 ^c^ ± 0.00	0.00 ^c^ ± 0.00	14.91 ^a^ ± 0.95	1.78 ^b^ ± 0.42
cycC19:0,*cis-*10,11 (lactobacillic/*cis-*11,12-methyleneoctadecanoic)	2.14 ^a^ ± 0.52	6.15 ^b^ ± 0.93	0.00 ^c^ ± 0.00	0.00 ^c^ ± 0.00	9.83 ^d^ ± 1.69	29.56 ^e^ ± 3.23
18:2,*cis-*9,*trans-*11 (conjugated octadecadienoic)	1.42 ^a^ ± 0.33	2.98 ^b^ ± 0.24	2.36 ^b^ ± 0.24	2.06 ^b^ ± 0.08	1.13 ^a^ ± 0.16	0.21 ^c^ ± 0.13
C18:2, CLA_1 (conjugated octadecadienoic)	0.13 ^a^ ± 0.02	0.00 ^b^ ± 0.00	0.20 ^c^ ± 0.03	0.16 ^c^ ± 0.01	0.11 ^a^ ± 0.04	0.00 ^b^ ± 0.00
18:2,*trans-*10,*cis-*12 (conjugated octadecadienoic)	0.77 ^a^ ± 0.20	0.00 ^b^ ± 0.00	2.09 ^c^ ± 0.32	1.95 ^c^ ± 0.28	1.21 ^d^ ± 0.14	0.00 ^b^ ± 0.00
C18:2, CLA_2 (conjugated octadecadienoic)	0.09 ^a^ ± 0.03	0.00 ^b^ ± 0.00	0.19 ^c^ ± 0.03	0.19 ^c^ ± 0.02	0.11 ^a^ ± 0.01	0.00 ^b^ ± 0.00
C18:2, CLA_3 (conjugated octadecadienoic)	0.02 ^a^ ± 0.03	0.19 ^b^ ± 0.01	0.15 ^c^ ± 0.04	0.13 ^c^ ± 0.03	0.10 ^a,b^ ± 0.01	0.00 ^d^ ± 0.00
C18:2, CLA_4 (conjugated octadecadienoic)	0.85 ^a^ ± 0.14	0.00 ^b^ ± 0.00	1.49 ^c^ ± 0.23	1.34 ^c^ ± 0.11	1.09 ^b^ ± 0.12	0.00 ^d^ ± 0.00
ratio cycC19:0,*cis-*9,10/cycC19:0,*cis-*10,11	7.30	0.12	0.00	0.00	1.52	0.06
ratio C18:1,*cis-*9/C18:1,*cis-*11	7.38	0.05	31.80	25.51	2.47	0.05
∑ unsaturated acids	69.86	73.99	82.58	88.26	46.01	34.86
∑ saturated acids	11.12	18.72	17.22	11.64	29.13	33.35
ratio unsaturated/saturated	6.28	3.95	4.80	7.58	1.58	1.05
∑ conjugated C18:2,*cis-*9,*cis-*12 acids	3.27	3.18	6.48	5.83	3.75	0.21
cis/trans	31.75	81.37	27.12	31.28	20.18	26.72
mono-/polyunsaturated	12.46	14.38	11.38	13.63	9.37	111.43
CLA/other conjugated fatty acids	0.77	0.06	0.86	0.88	1.25	0.00

Legend: Values are presented as mean ± standard deviation (n = 6). ^a,b,c,d,e^ Different superscript letters within a row indicate statistically significant differences (*p* < 0.05) between strains and/or growth conditions.

**Table 3:** Composition of the external standard (BAME) and identification parameters for each acid.

Fatty Acid	Acid Name	Retention Time (min)	ECL	Electron Ionisation
C11:0	undecanoic	15.466	11.000	74, 87, 143, 157, 200
C12:0	lauric/dodecanoic	17.655	12.000	74, 87, 143, 214
C13:0	tridecanoic	19.751	13.000	74, 87, 143, 185, 228
C14:0	myristic/tetradecanoic	21.757	14.000	74, 87, 143, 199, 242
C10:0,2OH	2-hydroxydecanoic	22.658	14.481	69, 83, 143, 228
C15:0,iso	iso-13-methyltetradecanoic	22.777	14.542	74, 87, 143, 213, 256
C15:0,anteiso	anteiso-12-methyltetradecanoic	23.084	14.701	74, 87, 143, 213, 256
C15:0	pentadecanoic	23.673	15.000	74, 87, 143, 213, 256
C16:0,iso	iso-14-methylpentadecanoic	24.647	15.541	74, 87, 143, 227, 270
C16:0	palmitic/hexadecanoic	25.505	16.000	74, 87, 143, 227, 270
C17:0,iso	iso-15-methylhexadecanoic	26.442	16.541	74, 87, 143, 241, 284
C16:1,*cis-*9	palmitoleic/hexadecenoic	26.485	16.565	69, 83, 96, 152, 236
C12:0,2OH	2-hydroxydodecanoic	26.636	16.653	69, 83, 97, 171, 230
C17:0	heptadecanoic	27.255	17.000	74, 87, 143, 241, 284
cycC17:0,*cis-*9,10	*cis-*9,10-methylenehexadecanoic	27.965	17.432	69, 74, 83, 97, 250
C18:0	stearic/octadecanoic	28.934	18.000	74, 87, 143, 255, 298
C12:0,3OH	3-hydroxydodecanoic	29.175	18.159	71, 74, 83, 103
C18:1,*trans-*9	elaidic/octadecenoic	29.492	18.351	69, 74, 83, 97, 123, 264
C18:1,*cis-*9	oleic/octadecenoic	29.705	18.486	69, 74, 83, 97, 123, 264
C14:0,2OH	2-hydroxytetradecanoic	30.221	18.797	69, 83, 97, 199
C19:0	nonadecanoic	30.545	19.000	74, 87, 143, 312
C18:2,*cis-*9,*cis-*12	linoleic/*cis-*9,cis12-octadecadienoic	31.015	19.307	97, 81, 95, 123, 294
cycC19:0,*cis-*9,10	dihydrosterculic/*cis-*9,10-methylene-octadecanoic	31.122	19.376	69, 74, 83, 97, 123, 278
C20:0	eicosanic	32.086	20.000	74, 87, 143, 326
C14:0,3-OH	3-hydroxytetradecanoic	32.592	20.318	71, 74, 103
C16:0,2-OH	2-hydroxyhexadecanoic	33.475	20.864	69, 83, 97, 227

**Table 4:** An exemplary fatty acid profile of lactobacilli biomass and identification parameters for each acid.

Fatty Acid	Acid Name	Retention Time (min)	ECL	Electron Ionisation
C10:0	caproic/decanoic	13.165	10.000	74, 87, 143
C12:0	lauric/dodecanoic	17.629	12.000	74, 87, 143, 157, 200
C14:0	myristic/tetradecanoic	21.722	14.000	74, 87, 143, 199, 242
C15:0,iso	iso-13-methyltetradecanoic	22.740	14.545	74, 87, 97
C15:0,anteiso	anteiso-12-methyltetradecanoic	23.035	14.698	74, 87, 143, 256
C15:0	pentadecanoic	23.626	15.000	74, 87, 143, 256
C 16:0	palmitic/hexadecanoic	25.477	16.000	74, 87, 143, 227, 270
C16:1,*trans-*9	palmitelaidic/*trans-*9-hexadecenoic	26.162	16.394	69, 83, 96, 152, 236
C16:1,*cis-*9	palmitoleic/*cis-*9-hexadecenoic	26.442	16.552	69, 83, 96, 152, 236
C12:0,2OH	2-hydroxydodecanoic	26.680	16.633	69, 83, 97
cycC17:0,*cis-*9,10	*cis-*9,10-methylenehexadecanoic	27.919	17.420	69, 74, 83, 250
C18:0	stearic/octadecanoic	28.869	18.000	74, 87, 143, 255, 298
C18:1	octadecenoic	28.935	18.040	69, 74, 83
C18:1,*trans-*6	petroselaidic/*trans-*6-octadecenoic	29.063	18.118	69, 74, 83
C18:1,*trans-*9	elaidic/*trans-*9-octa-decenoic	29.143	18.167	69, 74, 83, 97, 143
C18:1,*trans-*11	*trans-*vaccenic/*trans-*11-octadecenoic	29.436	18.343	69, 74, 83, 97, 264
C18:1,*cis*-6	petroselinic/*cis-*6-octadecenoic	29.538	18.404	69, 74, 83, 97, 143
C18:1,*cis-*9	oleic/*cis-*9-octadecenoic	29.660	18.477	69, 74, 83, 97, 123, 264
C18:1,*cis-*11	*cis-*vaccenic/*cis-*11-octadecenoic	29.808	18.565	69, 74, 83, 97, 123, 264
C18:2,*trans-*9,*trans-*12	linoelaidic/*trans-*9, *trans-*12-octadecadienoic	30.289	18.847	67, 81, 95
C18:2,*cis-*9,*cis-*12	linoleic/*cis-*9,*cis*-12-octadecadienoic	30.963	19.272	67, 81, 95, 294
cycC19:0,*cis-*9,10	dihydrosterculic/*cis-*9,10-methyleneoctadecanoic	31.071	19.343	69, 74, 83, 97, 123, 278
cycC19:0,*cis-*10,11	lactobacillic/*cis-*11,12-methyleneoctadecanoic	31.159	19.401	69, 74, 83, 97, 123, 278
18:2,*cis-*9,*trans-*11	conjugated octadecadienoic	32.868	20.492	67, 81, 95, 123, 294
C18:2, CLA_1	conjugated octadecadienoic	32.965	20.552	67, 81, 95, 123, 294
18:2,*trans-*10,*cis-*12	conjugated octadecadienoic	33.098	20.634	67, 81, 95, 109, 263, 294
C18:2_CLA_2	conjugated octadecadienoic	33.219	20.709	67, 81, 95, 123, 294
C18:2_CLA_3	conjugated octadecadienoic	33.323	20.773	67, 81, 95, 294
C18:2_CLA_4	conjugated octadecadienoic	33.631	20.961	67, 81, 95, 123, 294

## Data Availability

The authors confirm that the data supporting the findings of this study are available within the article.
